# Glycan profiling of the gut microbiota by Glycan-seq

**DOI:** 10.1038/s43705-021-00084-2

**Published:** 2022-01-05

**Authors:** Lalhaba Oinam, Fumi Minoshima, Hiroaki Tateno

**Affiliations:** 1 Cellular and Molecular Biotechnology Research Institute, National Institute of Advanced Industrial Science and Technology (AIST), Tsukuba Central 6, 1-1-1 Higashi, Tsukuba, Ibaraki 305-8566 Japan; 2 AMED-Prime, AMED, Tsukuba Central 6, 1-1-1 Higashi, Tsukuba, Ibaraki 305-8566 Japan

**Keywords:** Next-generation sequencing, Microbiome

## Abstract

Bacterial glycans modulate the cross talk between the gut microbiota and its host. However, little is known about these glycans because of the lack of appropriate technology to study them. In this study, we applied Glycan-seq technology for glycan profiling of the intact gut microbiota of mice. The evaluation of cultured gram-positive (*Deinococcus radiodurans*) and gram-negative (*Escherichia coli*) bacteria showed significantly distinct glycan profiles between these bacteria, which were selected and further analyzed by flow cytometry. The results of flow cytometry agreed well with those obtained by Glycan-seq, indicating that Glycan-seq can be used for bacterial glycan profiling. We thus applied Glycan-seq for comparative glycan profiling of pups and adult mice gut microbiotas. The glycans of the pups and adult microbiotas had significantly distinct glycan profiles, which reflect the different bacterial compositions of pups and adult gut microbiotas based on 16S rRNA gene sequencing.α2-6Sia-binders bound specifically to the pups microbiota. Lectin pull-down followed by 16S rRNA gene sequencing of the pups microbiota identified *Lactobacillaceae* as the most abundant bacterial family with glycans reacting with α2-6Sia-binders. The Glycan-seq system can reveal the glycan profile of the intact bacterial gut microbiota.

## Introduction

The microbiota of the digestive tract [1] is dominated by bacteria. It is estimated that 1000 species of commensal, symbiotic, and pathogenic bacteria are present in the gut microbiota [2, 3]. The gut microbiota plays vital roles in human health and disease conditions and is tightly regulated by the lifestyle, dietary habits, and health status of the host [4]. It interacts with the gut epithelium, including the different immune cells within it [5]. The gut microbiota may play regulatory roles in mood, anxiety, and cognition via the gut–brain axis [6], and an imbalance in the gut microbiota may cause gastrointestinal disorders [4, 7] and metabolic [8] and inflammatory diseases [9].

The surface of the bacteria is coated with an intricate network of glycans that act as an interface between mammalian hosts and their gut bacteria [10]. Gram-positive bacterial cells are enclosed by a single membrane covered by a thick peptidoglycan layer and lipoteichoic acids [11], whereas gram-negative bacteria are covered by two cell membranes (inner and outer membranes) separated by a periplasm containing a thin peptidoglycan layer; the outer membrane consists of lipopolysaccharides [12]. Both types of bacteria are often further enclosed by a diverse array of capsular polysaccharides [13]. Compared to mammalian glycans, the bacterial glycans display similarities and diversity with unique glycan structures [14]. In bacteria, almost all types of monosaccharides, including common monosaccharides like 6-deoxy-L-mannose and 6-deoxy-L-galactose, are present. In addition, monosaccharides unique to bacteria such as 3,6-di-deoxyhexoses and Kdo are also found [15, 16].

For high-throughput and -sensitive glycan profiling of bacterial glycans, lectin microarrays containing glycan-binding proteins (lectins) have been applied. Using the lectin microarrays, the glycosylation pattern of similar strains of pathogenic and non-pathogenic *E. coli* has been reported to be different [17]. Lectin microarray analysis also revealed the different glycan profiles within the same bacterial strain grown in different culture conditions [18] as well as temperature-sensitive glycan profiles in *Campylobacter jejuni* [19]. We also compared 16 different strains of *Lactobacillus casei* using lectin microarrays [20]. Interestingly, we found that bacterial cell surface glycans are different depending on the bacterial strains. These studies demonstrated that lectin-based glycan profiling is applicable to bacteria. However, there are several drawbacks in the lectin microarray analysis of bacteria, including the following: (1) A large number of cells (1 × 10^8^–5 × 10^9^ cells/well) are required for the analysis. (2) Bacteria bound to the lectin microarray are easily released by washing steps meant to remove unbound bacteria. Thus, the results of this analysis may be difficult to reproduce. (3) The analysis requires fluorescently labeled bacterial cells, and different species of bacteria may differ in their fluorescence. As the gut microbiota consists of various bacterial populations, labeling all bacterial populations at the same level of fluorescence is difficult. Hence, the lectin microarray has never been applied to the analysis of the gut microbiota. Thus, despite playing an essential role in bacterial cross talk with the host, bacterial glycans in the gut microbiota remain poorly understood mainly because of insufficient analysis method.

We recently developed a highly multiplexed glycan profiling method called Glycan-seq, which analyzes bulk and single cells using DNA-barcoded lectins and next-generation sequencing [21]. In this study, we applied Glycan-seq to analyze the gut microbiota of mice without performing any prior bacterial culturing and fluorescence labeling. First, we evaluated the applicability of Glycan-seq for bacterial profiling using the cultured representatives of gram-positive and gram-negative bacteria. We then used Glycan-seq to analyze the glycan alteration on the gut microbiota of pups and adult mice. Further, 16S rRNA gene sequencing was performed to analyze the differences in the bacterial composition of the gut microbiotas in pups and adult mice.

## Methods

### Microbial culture

*Escherichia coli* (Migula) (ATCC 700926) was cultured overnight at 37 °C in M9 culture medium, whereas *Deinococcus radiodurans* (ATCC BAA-816) was also cultured overnight at 30 °C in TGY medium. The abundance and size of cells were analyzed using a particle counter (CDA 1000; Sysmex Corporation, Hyogo, Japan).

### Mice

Pups (14–20 days old) and adult (12 months old) C57BL/6J mice were used in this study. The mice were derived or purchased from Charles River Laboratories and Japan SLC (Shizuoka, Japan). Male mice were used for all the experiments. The mice were housed under specific pathogen-free conditions in the Laboratory Animal Resource Center at the University of Tsukuba, Japan.

### Fecal sample collection and microbiota isolation

Mice were placed inside an autoclaved cage for 30–60 min, and the excreted feces were collected using sterilized forceps. The collected feces were frozen at −20 °C until use. The mouse fecal microbiota was isolated using the density gradient method [22]. Briefly, ~20 mg of feces was homogenized in 0.5 mL phosphate-buffered saline (PBS) at 4 °C by shaking at 750 rpm overnight. After homogenization, the supernatant was collected and transferred to the top of a Nycodenz solution (80% w/v in water; Cosmo Bio Co., Ltd., Tokyo, Japan). The solution was then centrifuged at 10,000 × *g* for 40 min at 4 °C. The middle layer containing the microbiota was collected and further washed with PBS. The numbers and sizes of bacterial cells from each sample were quantified using a particle counter (CDA-1000; Sysmex Corporation, Hyogo, Japan).

### Preparation of DNA-barcoded lectins

Lectins were conjugated to the DNA oligonucleotide as previously described [21]. Briefly, 100 μg of each lectin was dissolved in 100 μL of PBS mixed with dibenzocyclooctyne-N-hydroxysuccinimidyl ester (DBCO-NHS) (Funakoshi Co., Ltd., Tokyo, Japan) at 10 times the molar amount and then incubated in the dark for 1 h at 20 °C. DBCO-NHS was inactivated by adding 10 μL of 1 M Tris and incubating the mixture in the dark for 15 min at 20 °C. The excess DBCO-NHS was removed using Sephadex G-25 desalting columns (GE Healthcare Japan Co., Tokyo, Japan). The DBCO-labeled lectin product (100 μg/mL) was mixed with 5′-azide-modified DNA oligonucleotides (Integrated DNA Technologies, KK, Tokyo, Japan) at ten times the molar amount. The conjugated lectin-DNA oligonucleotide was purified by removing unbound nucleotides and selecting only the lectins with the glycan-binding affinity, which was achieved by affinity chromatography using the appropriate sugar-immobilized Sepharose 4B-CL (GE Healthcare Japan Co., Tokyo, Japan) based on the glycan-binding specificity of each lectin.

### Glycan-seq

Bacterial cells (1 × 10^7^) were suspended in PBS containing 1% bovine serum albumin (BSA) and incubated with 39 DNA-barcoded lectins at a final concentration of 0.5 μg ml^−1^ at 4 °C for 1 h. The cells were washed three times with 1 mL of PBS/BSA to liberate oligonucleotides after which it was diluted ten times (1 × 10^6^) and then were UV-irradiated at 365 nm, 15 W, for 15 min using a UVP Blak-Ray XX-15L UV Bench Lamp (Analytik Jena, Kanagawa, Japan). The liberated oligonucleotides were then amplified using NEBNext Ultra II Q5 (New England BioLabs Japan Inc., Tokyo, Japan), i5-index, and i7-index primers containing cell oligonucleotide sequences. PCR reactions were performed as follows: 1 cycle of denaturation for 45 s at 98 °C; 20 cycles of denaturation for 10 s at 98 °C, followed by 50 s at 65 °C; and 1 cycle of extension for 5 min at 65 °C. The PCR products were then purified using the Agencourt AMPure XP Kit (Beckman Coulter, Inc., Tokyo, Japan), following the manufacturer’s protocol. The size and quantity of the PCR products were analyzed using MultiNA (Shimadzu Co., Kyoto, Japan). The PCR products (4 nM from every sample) were treated with the MiSeq Reagent Kit v2 (50 cycle format; Illumina KK, Tokyo, Japan) and sequenced using the MiSeq Sequencer (26 bp, paired-end) (Illumina KK, Tokyo, Japan).

### Analysis of Glycan-seq data

The DNA barcodes derived from lectins were directly extracted from the reads in FASTQ format. The number of DNA barcodes bound to each cell was counted using a barcode DNA counting system (Mizuho Information & Research Institute, Inc., Tokyo, Japan) [21]. The first three bases in each read were removed to better match the DNA barcode sequence. In cases of mismatch, we allowed a maximum of two mismatches in the flanking region and one mismatch in the middle region. The total number of each of the DNA barcodes was divided by the total number of lectin barcodes and expressed as a percentage (%) for each lectin. Statistically significant levels of lectins in the Glycan-seq were evaluated using abundance analysis of compositional data adapted from the Analysis of Compositions of Microbiomes (ANCOM) [23], with Holm-Bonferroni multiple hypothesis testing corrections. Statistical significant lectins *p* < 0.05 were selected.

### Flow cytometry analysis

Approximately 1 × 10^7^ cells of *E. coli* and *D. radiodurans* were incubated with 10 μg of R-phycoerythrin-conjugated lectins for 1 h on ice. For the inhibition assay of the lectins, 0.2 M of N-Acetyl-D-glucosamine (GlcNAc)(Nacalai Tesque, Japan) was used for rABA and rSRL. Similarly, 0.2 M of D-(+)-mannose (21306-15, Nacalai Tesque, Japan) was used for the inhibition of rGRFT and rBanana. The sugars were added during incubation of the lectins. For the mannosidase treatment of cultured *D. radiodurans*, ~1 × 10^7^ bacterial cells were incubated at 37 °C for 8 h with agitation (750 rpm) in the presence of 4U of α-1, 2, 3, 6 mannosidase (New England BioLabs Japan Inc., Tokyo, Japan), in a total volume of 20 μL of 1% BSA/PBS, 2 μL of GlycoBuffer 4 (10X) and 1 μL of Zinc (10X). BSA-conjugated lectin was used as a negative control. Flow cytometry data were acquired on a CytoFLEX System (Beckman Coulter, Inc., Brea, CA) and analyzed using the FlowJo software v10.6 (BD, Franklin Lakes, NJ).

### Microbial DNA extraction from mouse feces

Genomic DNA was isolated from the microbial fraction collected from mouse feces (as described above) by a bead-beating method implemented using the ISOSPIN Fecal DNA Kit (Nippon Gene Co., Ltd, Japan). The isolated DNA was eluted in 50 μL TE buffer (pH 8.0) provided in the kit.

### 16S rRNA gene sequencing

Sequencing libraries were prepared from the V3–V4 hypervariable region of 16S rRNA gene, following the protocol entitled “16S Metagenomic Sequencing Library Preparation” from Illumina [24]. The V3–V4 hypervariable region of 16S rRNA gene was amplified using the following primers: forward: 5′-TCGTCGGCAGCGTCAGATGTGTATAAGAGACAGCCTACGGGNGGCWGCAG-3′; reverse: 5′-GTCTCGTGGGCTCGGAGATGTGTATAAGAGACAGGACTACHVGGGTATCTAATCC-3′). The 25 μL PCR reaction was performed using a KAPA HiFi HotStart ReadyMix (Roche Applied Science, Upper Bavaria, Germany) and contained 1 μL of extracted fecal microbial DNA and 1 μM of each primer. The reaction cycles consisted of initial denaturation at 98 °C for 2 min; followed by 25 cycles of denaturation at 98 °C for 15 s, annealing at 56 °C for 30 s, and elongation at 72 °C for 30 s; and a final elongation at 72 °C for 5 min.

Next, a second PCR was performed using Illumina index primers and the following reaction cycle: initial denaturation at 95 °C for 3 min; followed by 8 cycles of denaturation at 95 °C for 30 s, annealing at 55 °C for 30 s and elongation at 72 °C for 30 s; and a final elongation at 72 °C for 5 min. The amplicons were quantified using MultiNA (Shimadzu, Japan), a microchip electrophoresis system for DNA/RNA analysis. The amplicons were sequenced using the Illumina MiSeq 2 × 250 bp platform with a MiSeq Reagent Nano Kit V2 (Illumina KK, Tokyo, Japan).

### 16S rRNA gene sequence analysis

The raw sequence reads were analyzed using QIIME2 (2020.8) [25]. The reads were first demultiplexed; then, the DADA2 [26] plugin was used for quality control, read trimming, and assembly. Trimming took into consideration the information needed to merge the paired reads. Amplicon sequence variants (ASVs) were generated by DADA2 analysis, which were then classified to family and genus levels using the q2-feature-classifier [27], a Naïve Bayes machine learning classifier plugin in the QIIME2. Operational taxonomic units (OTUs) were generated by the RESCRIPt QIIME2 plugin running a feature classifier trained on the V3–V4 region of the 16S rRNA gene using a preformatted SILVA 138 reference database [28, 29]. An equal sampling depth of 10,000 was selected for every sample for assessing the diversities. α-diversity was measured by Faith’s phylogenetic diversity (PD) metrics, and significance (*p* < 0.05) was statistically calculated using Kruskal-Wallis (pairwise) analysis. Using principal coordinate analysis from the UniFrac metrics analysis, β-diversity was calculated [30].

### Microbe isolation by lectin-coated beads

SSA and TJAI lectins were labeled with biotin and used at a concentration of 1 μg/µL. The labeled lectins were incubated with streptavidin-conjugated Dynabeads (Thermo Fisher, Waltham, Massachusetts, USA) in a shaker set at 1400 rpm at 4 °C for 1 h. The conjugated beads were washed; then, 2 × 10^7^ microbial cells from pups and adult mice samples were incubated with the beads in a shaker at 700 rpm at 4 °C overnight. The bound microbes were isolated using a magnetic stand and eluted with 0.2 M lactose.

### Sialidase treatment of bacterial cells isolated from pups microbiota

1 × 10^7^ bacterial cells isolated from pups were incubated with 1 µL of sialidase (0.01 U, Roche Applied Science, Upper Bavaria, Germany) and incubated at 37 °C for 1 h. After the treatment, flow cytometry analysis was performed with 10 μg of R-phycoerythrin-conjugated lectins for 1 h on ice. For positive control of sialidase treatment, beads coated with 6′-sialyllactose-PAA-biotin (Glycotech, Gaithersburg, MD) was used. For flow cytometry analysis, BSA-conjugated lectin was used as a negative control. Flow cytometry data were acquired on a CytoFLEX System (Beckman Coulter, Inc., Brea, CA) and analyzed using the FlowJo software v10.6 (BD, Franklin Lakes, NJ). Statistical significance of the mean fluorescence intensity was determined by a two-tailed Student’s *t* test following a normal distribution.

## Results

### Glycan profiling of the gut microbiota by Glycan-seq

**Fig. 1:**
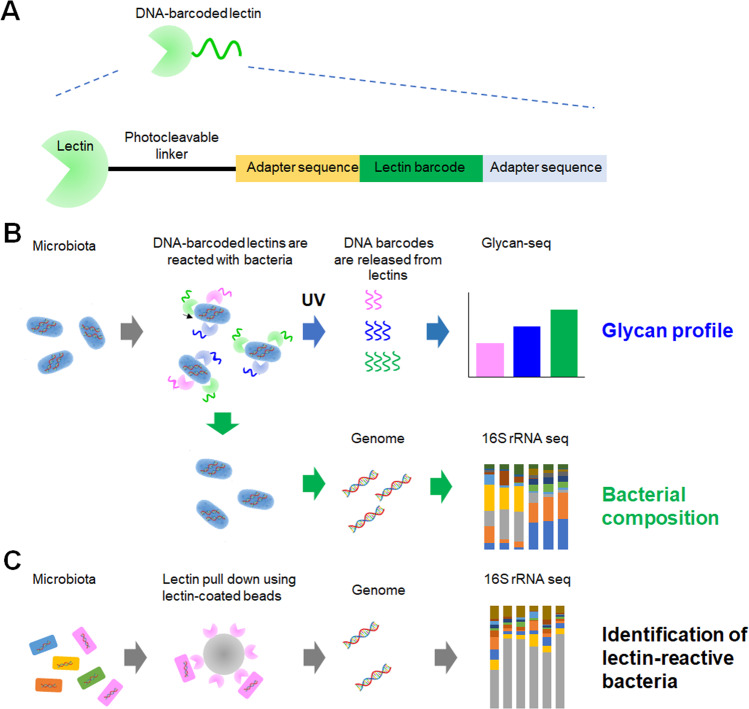
Glycan profiling of the gut-microbiota by Glycan-seq. **A** Schematic representation of a DNA-barcoded lectin. **B** Experimental workflow of the Glycan-seq and 16S rRNA sequencing of the gut-microbiota. **C** Schematic representation of lectin pull-down followed by 16S rRNA sequencing to identify lectin-reactive bacteria.

We aimed to develop a strategy for glycan profiling of the intact gut microbiota without prior fluorescence labeling using Glycan-seq [21]. Cultured bacterial cells were incubated with DNA-barcoded lectins, which, upon binding, released their DNA barcodes after UV exposure, because lectins were conjugated with DNA barcodes via a photocleavable linker (Fig. 1). The lectins used in this study cover a wide range of glycan structures, including sialylated, galactosylated, GlcNAcylated, mannosylated, and fucosylated glycans (Table S1). The released DNA barcodes were recovered, amplified, and analyzed by next-generation sequencing. The number of each DNA barcode was divided by the total number of lectin barcodes and expressed as percentage (%) values for each lectin. The microbiotas obtained from the pups and adult mice were also analyzed by 16S rRNA gene sequencing to identify the populations of bacteria.

### Glycan-seq of the cultured bacteria

**Fig. 2:**
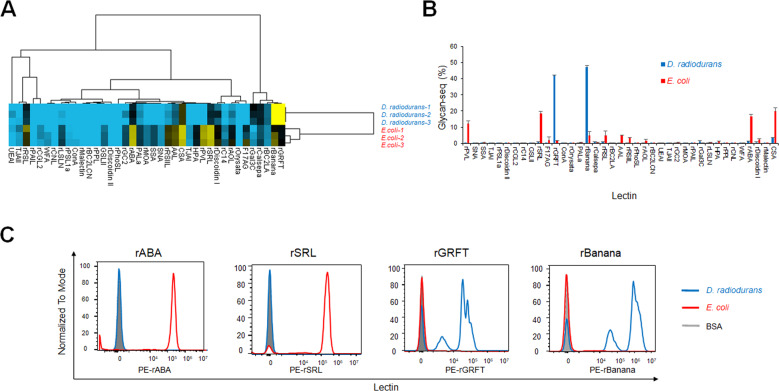
Glycan profiling of the cultured bacteria by Glycan-seq. **A** Hierarchical cluster analysis of *D. radiodurans* and *E. coli* using Glycan-seq data. **B** Graphical representation of Glycan-seq intensity data of *D. radiodurans* and *E. coli*. **C** Validation of GlcNAc-binders (rABA and rSRL) and mannose-binders (rGRFT and rBanana) identified by Glycan-seq using flow cytometry. Blue: *D. radiodurans*; red: *E. coli*.

We first evaluated whether Glycan-seq can be used to profile the glycans of cultured gram-positive *D. radiodurans* and gram-negative *E. coli* (Fig. 2, Table S2). Bacterial cells (1 × 10^7^) were incubated with DNA-barcoded lectins, and the DNA barcodes that were released from 1 × 10^6^ bacterial cells by UV irradiation were counted by sequencing. The resulting lectin binding signals were first analyzed by the hierarchical cluster analysis (Fig. 2A). The two types of bacteria were clearly separated into two different clusters, where the *x*-axis shows the lectins used and the *y*-axis shows the bacterial species. Several lectins differentially bound to *D. radiodurans* and *E. coli* (Fig. 2A and B). Among them, mannose-binders (rGRFT and rBanana) reacted at significantly higher intensities to *D. radiodurans*, whereas GlcNAc/Galβ1-3GalNAc-binders (rABA, rSRL, rPVL) exhibited higher intensities to *E. coli* (Fig. 2B).

### Validation of the results obtained by Glycan-seq using flow cytometry

We validated the results of lectin binding to *D. radiodurans* and *E. coli* obtained by Glycan-seq using flow cytometry analysis, which is considered the gold standard. By statistical analysis of the compositional data, twelve lectins were selected to show significantly different signals with *p* < 0.05 between the two different types of bacteria (Table S3). Based on the signal intensity (average intensity for positive cells, >15) obtained from Glycan-seq, we selected the following four lectins for flow cytometry analysis (Fig. 2B and Table S3): GlcNAc/Galβ1-3GalNAc-binders (rABA and rSRL) that generated higher signals in *E. coli* and mannose-binders (rGRFT and rBanana) that generated higher signals in *D. radiodurans*.

Flow cytometry using fluorescently labeled GlcNAc/Galβ1-3GalNAc-binders (rABA, rSRL) generated a higher peak signal in *E. coli*, whereas similar analysis using mannose-binders (rGRFT and rBanana) generated a higher peak signal in *D. radiodurans* (Fig. 2C). Thus, the results of flow cytometry agreed with those obtained by Glycan-seq (Fig. 2B). Taken together, these results indicate that bacterial glycan profiles generated by Glycan-seq can be recapitulated by flow cytometry analysis.

**Fig. 3:**
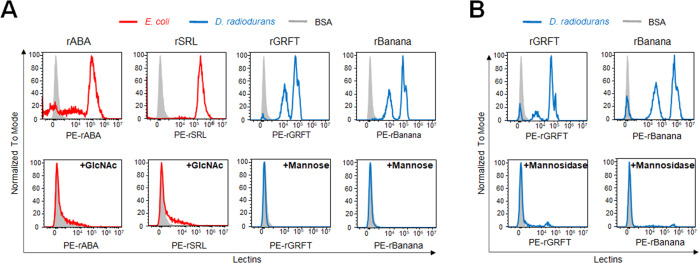
Inhibitory effect of monosaccharides and glycosidases on the lectin binding to the cultured bacteria. **A** *E. coli* and *D. radiodurans* were incubated with fluorescence-labeled GlcNAc-binders (rABA and rSRL) or mannose-binders (rGRFT and rBanana) in the absence (*top panel*) or presence (*bottom panel*) of monosaccharide inhibitors (GlcNAc for rABA and rSRL; mannose for rGRFT and rBanana). **B** *D. radiodurans* was incubated with fluorescence-labeled mannose-binders (rGRFT and rBanana) without (*top panel*) or with treatment of mannosidase (*bottom panel*). Blue: *D. radiodurans*; red: *E. coli*.

To evaluate whether the lectin binding to the cultured bacteria is due to the glycan-binding specificity of lectins, we performed a sugar inhibition assay in flow cytometry. The cultured bacteria were incubated with fluorescence-labeled lectins in the absence or presence of monosaccharide inhibitors and the inhibitory effect was evaluated by flow cytometry. For *E.coli* binding lectins (rABA and rSRL) with specificity to GlcNAc/Galβ1-3GalNAc, GlcNAc was used as a monosaccharide inhibitor, while mannose was used for *D. radiodurans* binding lectins (rGRFT and rBanana) with mannose-binding specificity. The addition of GlcNAc abolished the binding of rABA and rSRL to *E.coli*, while mannose eliminated the rGRFT and rBanana binding (Fig. 3A). Next, to further verify the glycan modification present in the bacterial cell surface, we performed mannosidase treatment to remove mannose modification in *D. radiodurans*, and the binding of mannose-binders (rGRFT and rBanana) was analyzed by flow cytometry. The mannosidase treatment abolished the binding of the mannose-binders to *D. radiodurans* (Fig. 3B). Taken together, these results demonstrate that the binding activity of mannose-binders and GlcNAc-binders to the cultured bacteria is due to the lectin binding specificity to mannose- and GlcNAc-modified glycans expressed in bacteria, respectively.

### Glycan-seq of the gut microbiota from pups and adult mice

**Fig. 4:**
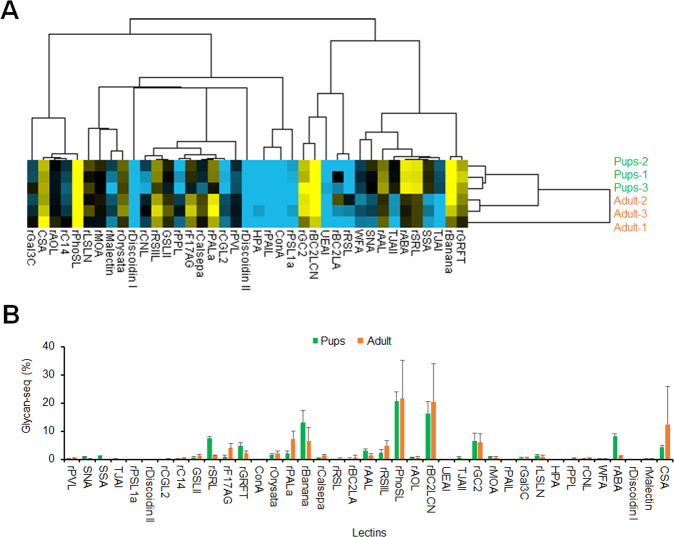
Glycan profiling of the gut microbiota of pups and adult mice. **A** Hierarchical clustering analysis of the gut microbiota of pups and adult mice using Glycan-seq data. **B** Graphical representation of the Glycan-seq data of the gut microbiota of pups and adult mice.

As Glycan-seq was applicable for both gram-positive and gram-negative bacteria, we used this approach to profile the gut microbiota of pups (preweaned) and adult (weaned) mice. Previous studies have shown that the composition and diversity of the gut microbiota change with age [31]. Bacterial cells were prepared from the fecal microbiota of pups (preweaned, 14–20 days old) and adult (weaned, 12 months old) mice (each *n* = 3), and the numbers and sizes of cells are shown in Table S4. The average number of bacterial cells recovered from the feces of pups mice was 1.6 × 10^10^ cells/g, which showed an average diameter size of 1.2 μm, whereas that from adult mice was 1.8 × 10^10^ cells/g with an average diameter of 0.97 μm. The fecal microbiotas (1 × 10^7^ cells) of pups and adult mice were then subjected to Glycan-seq, followed by hierarchical cluster analysis (Fig. 4, Table S5). The gut microbiotas of pups and adult mice were separated into two clusters based on Glycan-seq (Fig. 4A), demonstrating that the gut microbiotas of pups and adult mice have distinct glycan profiles. Several lectins showed distinct binding signals to pups and adult mice (Fig. 4B). Statistical analysis on the lectin intensity data obtained from Glycan-seq reveals four lectins that significantly differentiated the pups and adult microbiotas (Table S6). These lectins are α2-6Sia-binders (SSA, TJAI, SNA) and Galβ1-3GalNAc/GlcNAc-binders (rABA) (Table S6), all of which were detected at higher levels in the pups microbiota. Nevertheless, our data suggest that our newly developed Glycan-seq technology successfully profiled the glycan of the gut microbiota.

### Differences in the composition of the gut microbiota of pups and adult mice

**Fig. 5:**
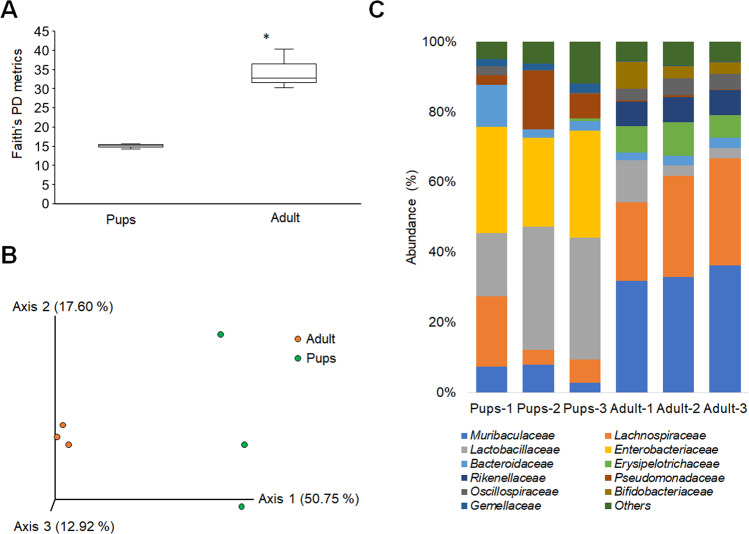
Comparison of the bacterial abundance between the pups and adult mice microbiota. **A** Boxplot of Faith’s phylogenetic diversity (PD) metrics analysis for the α-diversity. Statistical significance (*p* < 0.05) is denoted with an asterisk (*) calculated by Kruskal-Wallis pairwise analysis. **B** β-diversity analysis by principal coordinates analysis (PCoA) of unweighted UniFrac distance. **C** Stacked bar plot showing the taxonomy of the differential bacterial abundance in the pups and adult mice microbiota obtained from each mouse by 16S rRNA sequencing (*n* = 3 for each age group). Each colored bar plot indicates the family of bacteria identified, and for clarity, only the most abundant 11 families are shown, and the remaining are shown as others.

Based on 16S rRNA sequencing, the α- and β-diversity of pups and adult gut microbiota differed (Fig. 5A and B). This result is similar to that of a previous study [32] that reported differing compositions of the gut microbiota of pups and adult mice. The analysis of the ASVs of the metagenomic data of 16S rRNA gene sequences using QIIME2 [25] shows family-level differences between pups and adult microbiotas. Specifically, the relative abundance levels of *Lactobacillaceae*, *Enterobacteriaceae*, *Pseudomonadaceae*, and *Gemellaceae* were higher in the pup’s microbiota. In contrast, *Rikenellaceae*, *Erysipelotrichaceae*, *Muribaculaceae*, *Bifidobacteriaceae*, and *Lachnospiraceae* were higher in adult microbiota (Fig. 5C). Therefore, these differences in the gut microbiota diversity may explain the different glycan profiles of pups and adult microbiotas, as revealed by Glycan-seq analysis.

### Identification of sialylated bacteria in the gut microbiota of pups mice

**Fig. 6:**
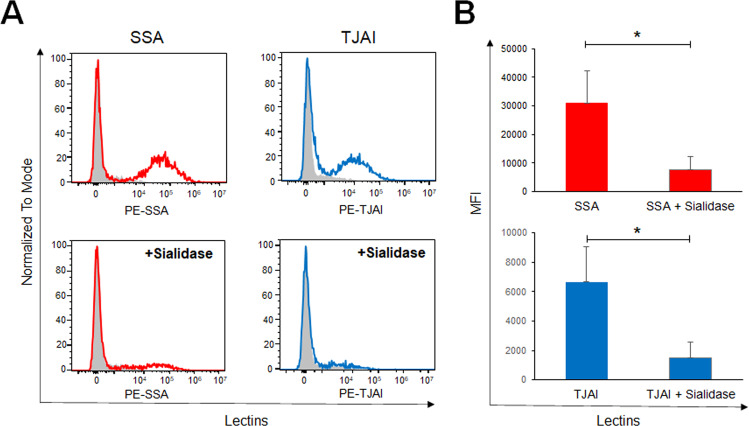
Sialidase treatment eliminates the binding of the α2-6Sia-binders to the gut microbiota from pups. **A** Fluorescence-labeled α2-6Sia-binders (SSA and TJAI) were incubated with the gut microbiota prepared from pups feces without (*top panel*) or with treatment of sialidase (*bottom panel*) and analyzed by flow cytometry. **B** Quantification of mean fluorescence intensity (MFI) obtained by flow cytometry. Data are shown as means ± SD. Statistical analysis was performed using the two-tailed unpaired Student’s *t* test. *p < 0.05.

The signal intensities of α2-6Sia-binders (SSA, TJAI, SNA) were significantly higher in the microbiota of pups mice. We were thus interested in investigating which bacteria are covered with sialic acid (Sia), a monosaccharide that is primarily found at the nonreducing end of glycoconjugates in eukaryotes and is involved in a variety of cell–cell interactions and cell–molecule recognition [33]. Several species of pathogenic bacteria display Sia on their outer surfaces, which masks them from the host immune system [34]. First, we investigated whether the removal of Sia from the gut microbiota of pups inhibit the binding of the α2-6Sia-binders. The binding of the α2-6Sia-binders (SSA, TJAI) to the gut microbiota with or without treatment of α2-3,6,8,9 sialidase derived from *Arthrobacter ureafasiense* was analyzed by flow cytometory (Fig. 6). Remarkably, the sialidase treatment abolished the binding of α2-6Sia-binders (SSA, TJAI) to the gut-microbiota of pups, suggesting that these lectins were identifying Sia-modified glycans in the gut-microbiota.

Next, we used a lectin pull-down assay to determine which bacteria in the microbiota of pups mice react with α2-6Sia-binders. Bacterial cells isolated from pups and adult mice were incubated with magnetic beads coated with α2-6Sia-binders (SSA and TJAI). The average number of cells recovered from the pups microbiota was 2.5 × 10^6^ cells/g, with an average diameter of 1.1 μm, whereas only approximately one-hundredth of bacterial cells (an average of 1.9 × 10^4^ cells/g with an average diameter of 0.6 μm) were obtained in the adult mice (Table S7). A large number of bacterial cells could be recovered from the microbiota of pups relative to adult mice, indicating that a higher number of bacterial cells modified with Sia are present in pups mice (Table S7).

**Fig. 7:**
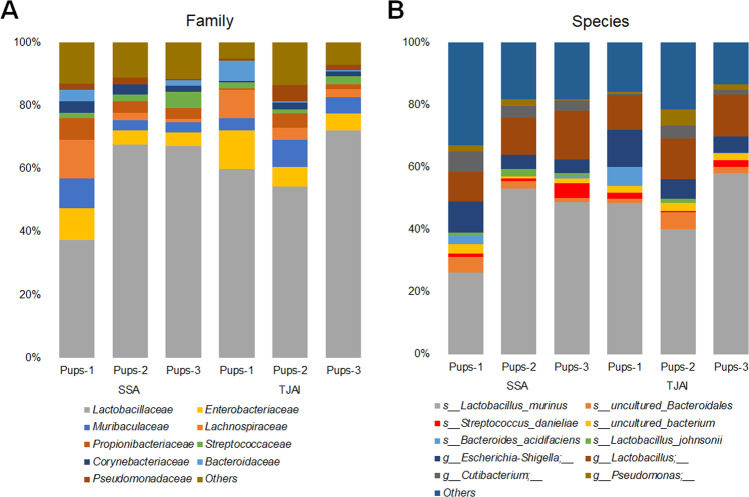
Family of bacteria reactive to α2-6Sia identified by lectin pull-down and 16S rRNA sequencing from the pups microbiota. Stacked taxa bar plot represents the **A** family **B** species of bacteria pull-downs by the α2-6Sia binders (SSA and TJAI). Each colored bar plot indicates the family or genus of bacteria identified, and for clarity, only the most abundant families or species are shown, and the remaining are shown as others.

The taxonomic groups of bacteria pulled down by the α2-6Sia-binders was then analyzed by 16S rRNA gene sequencing. The ASV analysis (using QIIME2) of the 16S rRNA gene sequences from the metagenomic data identified the phylogenetic distribution and relative abundance of bacteria pulled down by SSA and TJAI. The obtained results identified the families of bacteria reacted with α2-6Sia-binders in the pup’s microbiota (Fig. 7A). Both lectins detected mainly *Lactobacillaceae*, *Lachnospiraceae*, *Enterobacteriaceae*, and *Muribaculaceae* (Fig. 7A). At the genus level, these bacteria consisted mainly of *Lactobacillus*, *Pseudomonas*, *Escherichia-Shigella*, and *Streptococcus* (Fig. 7B). These results demonstrate that these bacterial families and genera modified with sialylated glycans might be present in the gut-microbiota of pups mice.

## Discussion

The genome and metabolome are the two major omics data acquired for the microbiota. In this sense, the information of the intact bacterial cell surface molecules without prior in vitro culturing, which directly mediate microbe–host interactions, could not be acquired. Actually, bacterial cell surfaces are coated with a diverse array of glycans that play pivotal roles in various biological processes. In particular, they mediate microbe–host interactions during the onset and development of infectious diseases and symbiotic interactions. However, our understanding of the glycan of the gut microbiota remains limited because of the lack of appropriate methods of analysis. In this study, we have developed a new sequencing-based technology that can analyze the glycan of bacteria in an intact form. The Glycan-seq technology offers several advantages: (1) Live bacterial cells can be analyzed without the need for prior culturing. (2) Fluorescence labeling of bacteria is not required. (3) A relatively low number of bacterial cells (~10^6^ cells) are required for the analysis. (4) Glycan profiles can be acquired using a conventional next-generation sequencer, the same instrument used for 16S rRNA gene sequencing.

The diversity of the gut microbiota of adult mice differed from that of pups mice. The relative abundance levels of *Lactobacillaceae*, *Enterobacteriaceae*, *Pseudomonadaceae*, and *Gemellaceae* were higher in pups than in adult microbiota, whereas those of *Rikenellaceae*, *Erysipelotrichaceae*, *Muribaculaceae*, *Bifidobacteriaceae*, and *Lachnospiraceae* were higher in adults than in pups microbiota. A study on calorie-restricted mice found reduced levels of *Lactobacillus*, which was negatively correlated with mice lifespan [35]. Furthermore, the abundance of *Lactobacillus murinus* is higher in calorie-restricted mice, and this species promoted anti-inflammation, which may play an important role during aging [36].

Several studies have sought to understand how the gut microbiota changes during aging and the biological significance of such changes [37]. The results from longitudinal studies on fecal samples from various individuals of different ages show age-related changes in the diversity and composition of the human gut microbiota [38, 39]. The composition of the gut microbiota of adults is unique, and the α-diversity of this microbiota increases with age, suggesting a correlation between the composition of the gut microbiota and physiological aging [39].

In this study, we report for the first time that the glycan of the gut microbiota changes during aging. Glycan-seq technology was able to profile the microbial glycans of pups and adult mice, and interestingly, α2-6Sia-binders reacted at significantly higher levels with the pups microbiota suggesting that the levels of sialylated bacteria decrease during aging. The bacterial families that reacted most with α2-6Sia-binders are *Lactobacillaceae*, *Lachnospiraceae*, *Enterobacteriaceae*, and *Muribaculaceae*. Previous findings that some *Lactobacillus* species express genes involved in the catabolism of Sia [34] are consistent with our results. The presence of Sia in *Lactobacillus* species has also been previously reported [40]. Several pathogenic bacteria such as enterohemorrhagic *Escherichia coli*, *Haemophilus influenzae*, *H. ducreyi*, *Pasteurella multocida*, *Neisseria gonorrhoeae*, *N. meningitidis*, *Campylobacter jejuni*, and *Streptococcus agalactiae* are well known to display Sia on their outer surfaces, which masks them from the host immune system. These bacteria have developed different mechanisms for obtaining Sia, including the *de novo* biosynthesis of Sia (*E. coli*, *N. meningitidis*), Sia scavenging (*N. gonorrhoeae*), and precursor scavenging (*H. influenzae*) [34]. One of the functions of Sia is the regulation of innate immunity by providing a mechanism of identifying self from nonself. However, various microbes have evolved a counter-mechanism that works by decorating the bacterial cell surface with similar Sia modifications [41]. Sia modified at the bacterial surface regulates the host immune system by interacting with Sia-binding immunoglobulin-type lectins (Siglecs) [42]. The presence of the Sia on the surface of bacteria also protects them against invading bacteriophage by blocking the relevant underlying receptors [43]. Therefore, the presence of Sia on the surface of bacteria in the gut microbiota of pups mice suggests that these microbes are protected from the host’s innate immune surveillance system and from bacteriophage, and their establishment proceeds from an initial colonization by microbes in the gut of pups mice [41].

Most bacteria obtain Sia by scavenging it from the surrounding environment [34]. Therefore, the glycans of bacteria cultured in vitro most probably differ from that of the same bacteria growing in the gut. Given this situation, Glycan-seq is useful because it can capture the glycan information of gut bacteria in situ, because it does not require prior culturing in vitro. There are limitations of the current method: (1) glycan-binding specificity of lectins to bacterial glycans is largely unknown; (2) the glycan profile of single bacterial cells cannot be obtained; and (3) the method is unable to determine the detailed structure of glycans.

## Conclusions

Our results suggest that the Glycan-seq method is an excellent choice for profiling the glycan of the gut microbiota. Our data provide important (and previously unknown) details about the changes in the glycan of the gut microbiota during aging. Glycan-seq analysis, in parallel with 16S rRNA gene sequencing, can identify the bacteria modified with Sia. It will be interesting to apply the Glycan-seq technology in future studies, seeking to understand how the glycan of the gut microbiota changes in response to dietary changes and disease development. Moreover, application of the Glycan-seq method to profile the glycan of a single bacterial cell, along with the bacterial identification, will help researchers understand the glycome architecture of the gut microbiota and its interaction with the host. In addition, our technology can also be applied to profile the glycans of other bacterial communities, such as those in the soil, deep ocean, and volcanic deposits.

## Availability of data and materials

All the data generated and analyzed have been included in the article or as Supplementary tables and files. The raw 16S rRNA amplicon sequencing data are deposited and publicly available from European Nucleotide Archive (ENA) at EMBL-EBI under accession number PRJEB45936.

## Supplementary information

Supplementary Tables 1–7.
